# Do Scholars Respond Faster Than Google Trends in Discussing COVID-19 Issues? An Approach to Textual Big Data

**DOI:** 10.34133/hds.0116

**Published:** 2024-02-26

**Authors:** Benson Shu Yan Lam, Amanda Man Ying Chu, Jacky Ngai Lam Chan, Mike Ka Pui So

**Affiliations:** ^1^ Department of Mathematics, Statistics and Insurance, The Hang Seng University of Hong Kong, New Territories, Hong Kong.; ^2^ Department of Social Sciences, The Education University of Hong Kong, New Territories, Hong Kong.; ^3^ Department of Information Systems, Business Statistics and Operations Management, The Hong Kong University of Science and Technology, New Territories, Hong Kong.

## Abstract

**Background:** The COVID-19 pandemic has posed various difficulties for policymakers, such as the identification of health issues, establishment of policy priorities, formulation of regulations, and promotion of economic competitiveness. Evidence-based practices and data-driven decision-making have been recognized as valuable tools for improving the policymaking process. Nevertheless, due to the abundance of data, there is a need to develop sophisticated analytical techniques and tools to efficiently extract and analyze the data. **Methods:** Using Oxford COVID-19 Government Response Tracker, we categorize the policy responses into 6 different categories: (a) containment and closure, (b) health systems, (c) vaccines, (d) economic, (e) country, and (f) others. We proposed a novel research framework to compare the response times of the scholars and the general public. To achieve this, we analyzed more than 400,000 research abstracts published over the past 2.5 years, along with text information from Google Trends as a proxy for topics of public concern. We introduced an innovative text-mining method: coherent topic clustering to analyze the huge number of abstracts. **Results:** Our results show that the research abstracts not only discussed almost all of the COVID-19 issues earlier than Google Trends did, but they also provided more in-depth coverage. This should help policymakers identify core COVID-19 issues and act earlier. Besides, our clustering method can better reflect the main messages of the abstracts than a recent advanced deep learning-based topic modeling tool. **Conclusion:** Scholars generally have a faster response in discussing COVID-19 issues than Google Trends.

## Introduction

With the availability of a huge amount of data, the rapid development of tools for mining big data has great potential for solving various problems related to COVID-19 [1–4]. Policymakers frequently face challenges when it comes to identifying health issues, establishing policy priorities, formulating suitable regulations, safeguarding citizens' freedoms, and promoting economic competition. Recent research has shown that the policymaking process can benefit from evidence-based practices [5,6]. This includes the use of empirical analyses informed by theory, data-driven decision-making, and a significant level of analytical capability. [7,8]. Advanced analytical techniques, such as mathematical modeling, can boost policymaking.

COVID-19 research work is widely acknowledged by experts from various domains as a valuable source of information for combatting the pandemic. However, the huge volume of research papers poses a challenge, as there are limited tools available for analysis. Timothy Sheahan, a virologist specializing in COVID-19, acknowledges the difficulty of keeping up with the ever-increasing number of scientific papers related to the disease and the novel coronavirus [9,10]. It is simply impossible for him to read through the extensive collection of available papers. Fortunately, a collaborative effort involving data scientists, software developers, and journal publishers is underway to address this issue. Major technology companies and the White House are actively working toward creating digital repositories^1^ of thousands of freely available papers that could potentially contribute to end the pandemic. Additionally, they are striving to develop data-mining and search tools to assist researchers to find information they require. Many teams are turning to advanced computational tools to extract data from databases like the COVID-19 Open Research Dataset (CORD-19). As an example, over 1,500 projects have been initiated by data scientists in response to calls from the White House to create tools that utilize CORD-19 to address inquiries from different institutions such as the National Academy of Sciences and the World Health Organization.

Current researches have predominantly used Google Trends (GT) indices to find out the relationships between different issues (e.g., oil market [11], vaccine [12], and lockdown [13]) and COVID infection/death rates [14–16], because GT could find out the most frequently searched words for the time and location selected and show what topics the public may be most interested in or responsive to during the selected period. Based on such search interest result, policymakers can design new policies to curb the pandemic. Despite the advantages, GT is not able to reflect factors that are important but have not been frequently searched, which may be covered and studied in academic research works. Therefore, a comparison between GT and such academic works may help present a more comprehensive picture of the pandemic and enable policymakers to identify key factors affecting the same and make appropriate policies accordingly.

To address these research gaps in the existing knowledge, we have 4 goals. First, we have proposed a new research framework to analyze the huge volume of textual data. In this work, we have applied the proposed framework to 2 worldwide bodies of information about COVID-19, using the keywords obtained from GT and the research abstracts (RAs) in the WHO COVID-19 research database. This new framework can be extended to other text-mining-related problems. Second, we have discussed the response times of the 2 worldwide information databases and explored the relationships between these 2 sets of textual information. The results provide a signal to policymakers about whether GT provides a faster or earlier warning in COVID-19-related issues than RAs do. The early signals and trends of the challenging factors provide important information to institutions and governments to make decisions, understand the complexity of problems and monitor citizens’ preferences [5,6]. Third, we have proposed a new text-mining method—the coherent topic clustering (CTC) method—to analyze the huge amount of information in the RAs. The greatest merit of the proposed methodology over recent advanced approaches is that it can generate topics that frequently appeared. The essential idea of the proposed method is to group the key phrases that frequently appear in the same abstracts. Because the key phrases frequently appear, they should be the major themes of the huge amount of text. This property is important because the number of abstracts is huge, and it is difficult for humans to dig through them and verify the obtained topics. Fourth, using the obtained key phrases and clusters, we can find the trends of different COVID-19 issues raised by researchers.

## Methods

### Data description

In this study, we used the textual data downloaded from GT and the RAs. The keywords from GT were obtained by searching for “related queries” of the 2 seed topics, namely, “COVID-19” and “COVID”, monthly from 2/1/2020 to 8/31/2022, in a worldwide setting. We considered all of the keywords with a positive “search interest relative to the highest point”, which is a numerical value ranging from 0 to 100. We had a total of 121 keywords after removing the repeated ones. Beyond that, we downloaded the RAs from the WHO COVID-19 Research Database^2^ and considered the English-language documents that were published from 2/1/2020 to 8/31/2022. A grand total of 400,158 documents were downloaded from the database, which serves as a comprehensive source of up-to-date international scientific research and knowledge on COVID-19. The primary objective of this database is to encourage collaboration among scientists and global health professionals worldwide, with the aim of accelerating the research and development process. Furthermore, it seeks to establish novel norms and standards for effectively containing the transmission of the coronavirus pandemic and providing assistance to those impacted by it.

### New research framework

**Fig. 1.**
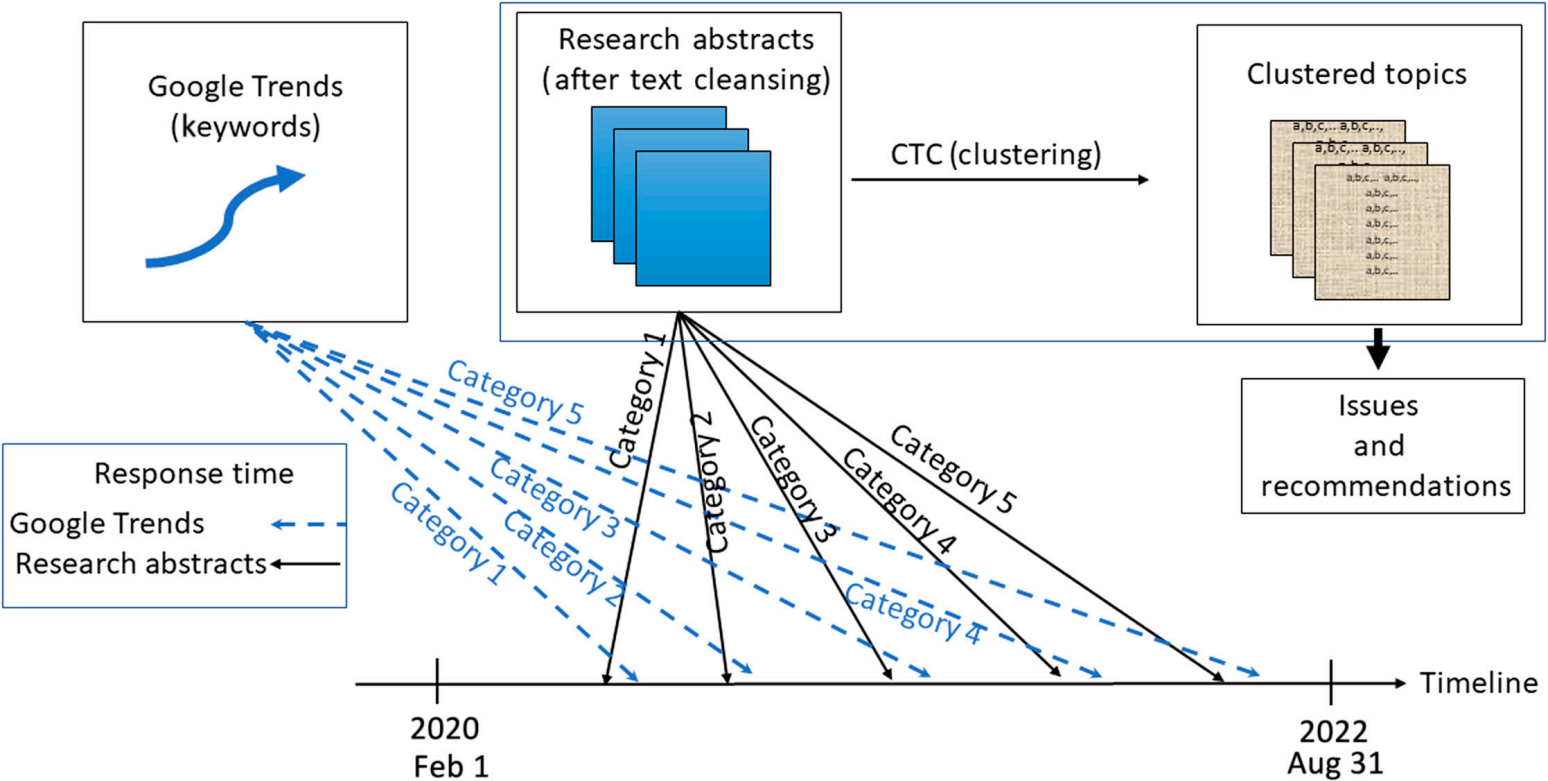
Infograph of the proposed research framework.

Our proposed research framework is shown as an infograph in Fig. 1. We grouped the GT keywords into different categories using a hierarchical clustering method (see the Supplementary Materials for more details), with the categories representing different policy aspects of COVID-19 from GT perspectives. Because GT does not provide any word associations among the keywords, we associated the keywords by examining their co-occurrences in the RAs database. The determination of the number of clusters involved identifying the most significant vertical difference between nodes in the dendrogram, and a total of 50 clusters were identified. After clustering, each category had a set of GT keywords. We then found the response times of these GT keywords in the RAs (see Response Time below) from 2/1/2020 to 8/31/2022. We then compared the response times of RAs and GT using bootstrapping statistics (see the Supplementary Materials for more details). We applied the proposed clustering method, namely, CTC (see below for more details), to more than 400,000 abstracts and examined the content of these RA results under different categories. The method grouped the noun phrases that appeared most frequently in the same abstracts—an approach that should generate the major themes of the abstracts. We then were able to explore the meanings of the groups of noun phrases and find the issues and recommendations that the researchers raised. Finally, we reviewed these associations and made recommendations, and we examined the impacts of the research results under different COVID statuses.

### Coherent topic clustering

The hierarchical clustering with complete link method was used to group the nouns or noun phrases of the selected abstracts together. First, we found the co-appearance of the keywords among the RAs and formed a very large matrix. We defined an appearance matrix, *A*_*i*,*k*_, where *i* was the *i*th noun phrase and *k* was the *k*th RA. The appearance matrix *A*_*i*,*k*_ = 1 if the *i*th noun phrase appeared in the *k*th RA. Otherwise, *A*_*i*,*k*_ = 0. The distance matrix of the clustering method was then obtained by finding the cosine similarity among the noun phrases along the RA. The formula is

$$\text{distance matrix}\left(i,j\right)=1-\frac{\sum_{k}A_{i,k}A_{j,k}}{\sqrt{\sum_{k}A_{i,k}^{2}}\sqrt{\sum_{k}A_{j,k}^{2}}},$$ (1)

where *i* and *j* are the *i*th and *j*th noun phrase. We finally used the distance threshold *θ* = 1 − 10^−10^ to cluster the phrases and decide the number of clusters. Because the distance threshold was close to one, the hierarchical clustering with complete link required that the distance between any 2 groups be close to one. In other words, the distances among all the elements of the same cluster were close to zero. This implied that the distances between any 2 noun phrases of the same clusters were distance matrix(*i*, *j*) = 0. The dot product between any appearance patterns was one. That is, *A*_*i*,*k*_ ≈ *A*_*j*,*k*_ for all *k*. In other words, when the *i*th noun phrase appeared, the *j*th noun phrase must appear as well. Therefore, this clustering method grouped phrases that appeared frequently in the same RA together. Moreover, this distance threshold estimated the number of clusters, and no user intervention was required. Because requiring the distances between any 2 clusters to be close to one could group data with similar appearance patterns together, any threshold value, such as *θ* = 1 − 10^−10^ or a similar value, gave similar clustering results.

**Cleansing for the RAs**

To ensure data cleanliness, standard text-cleaning procedures were executed, including the elimination of excessive white spaces, non-alphabetical characters, and stop-words, as well as the conversion of all words to lowercase. The python package, spacy^3^, was then used to retain the nouns or noun phrases as a subject or an object of a sentence. Lemmatization was applied to all nouns and phrases. Any nouns or nouns phrases that appeared fewer than 100 times throughout the 400,158 abstracts were discarded. Ultimately, a total of 4,664,063 noun phrases as subjects and 6,188,249 noun phrases as objects were found throughout all of the RAs. They were then used as input for the CTC method.

### Response time

For GT, because the keywords were downloaded on a monthly pattern, the first time a keyword was mentioned was response time throughout the study period (i.e., 2/1/2020 to 8/31/2022). For the RAs, we adopted the bootstrapping method to determine the response time of a keyword. We counted the occurrence of the keyword daily, and then we found the means using bootstrapping 10,000 times. If the 5th percentile of the means is greater than zero, the mention of the months is significant and thus it is the response time.

## Results

### GT keyword categorizations

**Fig. 2.**
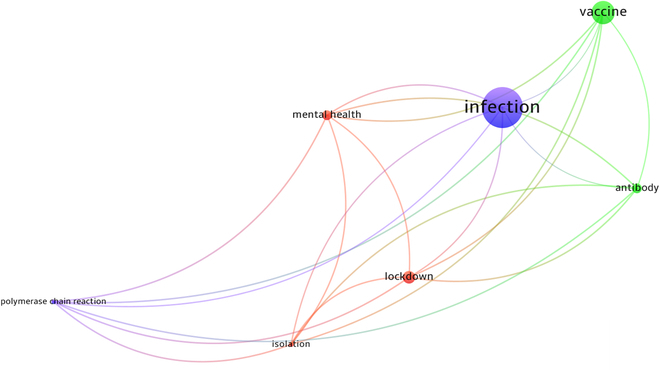
Network diagram of the GT keywords.

Figure 2 shows the associations of the GT keywords. Each vertex represents a keyword. The size of a vertex indicates the occurrence of the keywords in the RAs. An edge represents the number of co-occurrences between a pair of keywords. We treated the co-occurrences as a distance matrix and applied the hierarchical clustering to group keywords. Figure 2 shows the clustering results. The keywords belonging to the same clusters are represented by the same colors. We selected 3 sets of dominant nodes: (a) infection, polymerase chain reaction; (b) vaccine, antibody; (c) lockdown, mental health, isolation. It is noteworthy that the corresponding clusters had more than 3 nodes and 3 keywords. We only presented the nodes with the high occurrences. These groupings are similar to the classification of policy responses to COVID-19 developed by the Oxford COVID-19 Government Response Tracker^4^ (OxCGRT) [17,18]. The OxCGRT offers comprehensive international and temporal measurement to comprehend the progression of government responses throughout the entire duration of the pandemic. The OxCGRT categorizes policy responses into 4 main groups: (a) containment and closure policies, which encompass measures like school closures and movement restrictions; (b) health system policies, which include aspects such as COVID-19 testing protocols and emergency healthcare investments; (c) vaccine policies, which involve factors like prioritization lists, eligible groups, vaccination costs for individuals, and the presence of vaccine mandates; and (d) economic policies, which encompass measures such as income support for citizens and foreign aid provisions. With these categories, we refined the clusters of the keywords manually and grouped together the clusters with similar meanings. We then classified the clusters into the following categories: (a) containment and closure, (b) health systems, (c) vaccines, (d) economic, (e) country, and (f) others. It is noteworthy that the last 2 categories (i.e., country and others) are not listed in the OxCGRT, but their corresponding keywords appeared in the GT. Because of limited space, we put the discussions of (b) health systems, (e) country, and (f) others to Appendices M3, M4, and M5, respectively.

### Containment and closure

**Fig. 3.**
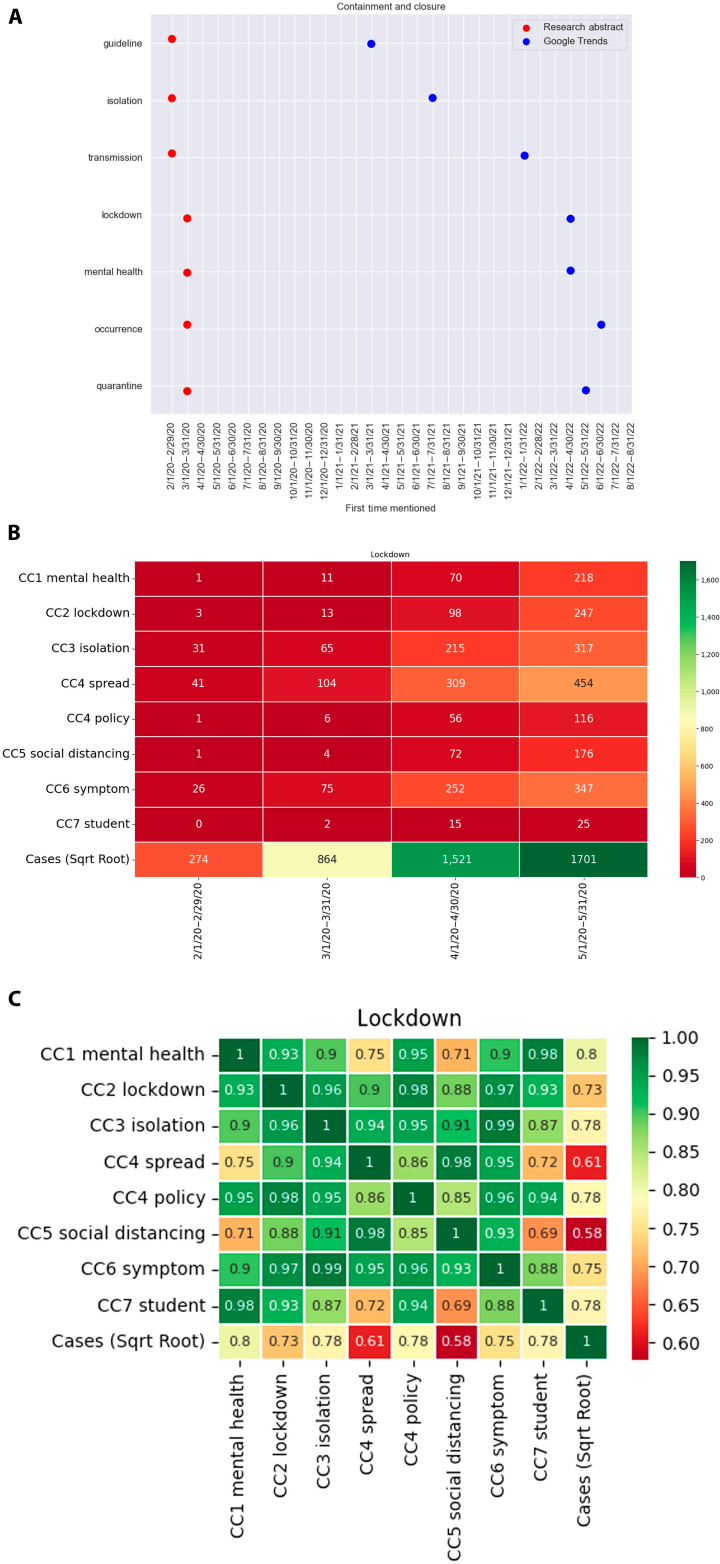
Analysis for the keywords/phrases under the category of containment and closure (CC). (A) Time plot for the keywords/phrases. The red dots represent the RAs, while the blue dots represent the GT. The *x*-axis shows the time periods, and the *y*-axis shows the keywords. (B) Occurrences of selected keywords/phrases and the number of reported COVID cases from 2/1/2020 to 5/31/2020. (C) Correlations among the selected keywords/phrases and the number of reported COVID cases of the entire study period (2/1/2020 to 8/31/2022).

To discuss the response times at which the keywords first appeared in the GT and RAs under this category, we considered the respective keywords: “guideline”, “isolation”, “transmission”, “lockdown”, “mental health”, “occurrence”, and “quarantine”, which could reveal different aspects of the containment and closure policies. The keywords “lockdown”, “isolation”, “quarantine”, and “guideline” can be viewed as the procedures of the policies; “transmission” and “occurrence” are the purpose of these policies; and “mental health” is one of the major issues of the policies. To observe whether the RAs mentioned these keywords earlier than the GT did, we considered the times at which the corresponding keywords were first mentioned. Figure 3A shows the time plot of the response time of the keywords of RAs and GT. It is very clear from the figure that the RAs mentioned these 7 keywords much earlier than GT did. The RAs discussed these topics in the first few months of the pandemic (that is, 2/1/2020 to 3/31/2020), whereas they were first discussed by GT at a much later date. For example, “lockdown” and “mental health” were first discussed from 4/1/2022 to 4/30/2022. We compared the first time that these keywords were mentioned in the RAs and GT using the paired bootstrapping mean and median differences (see the Supplementary Materials for more details). The 5th percentiles of the bootstrapping mean and median differences in the number of months of this category were 18.42 and 17, respectively. In other words, the research articles discussed these issues an average of 18.42 months (in terms of mean) and 17 months (in terms of median) earlier than the GT did, and the differences were significantly greater than zero—also indicating that the researchers discussed different aspects of containment and closure much earlier than GT searches did.

We next studied the contents of the RAs for the category of containment and closure. Here, we only considered the abstracts that contained at least one of the 7 GT keywords, and we found a total of 93,378 papers. We applied the proposed CTC method to these selected papers and obtained 2,329 clusters. For the sake of clarity, we hereafter denote these clusters as CTC clusters. We ranked each of the containment and closure CTC clusters according to its occurrences in the 93,378 papers, and we selected 7 of those CTC clusters for further discussion. Table 1 lists those 7 CTC clusters together with their noun phrases and rank scores. The rank score (i.e., the cluster’s noun phrases’ occurrence in the selected papers) was the highest for CTC cluster CC1, which comprised keywords that focused on mental and physical health. The next highest rank score was that of CTC cluster CC2, with keywords that focused on lockdowns, at 4,488, and the rank scores of the remaining 5 were all above 2,000. Thus, we see from these ranks that the themes of the papers were about mental and physical health and also about the impact and implementation of lockdown measures. Among these CTC clusters, we can see that each CTC cluster primarily represented one of the 3 aspects of containment and closure— the procedures, purpose, and major issues of the policies. The main themes of CTC clusters CC1, CC6, and CC7 were mental health, physical health, and depression and anxiety, with cluster CC7 representing papers that discussed the mental health issues of students, whereas CTC cluster CC4 included papers about how containment and closure policies helped stop the transmission of the virus—that is, the purpose of the policy. Clusters CC3 and CC5 represented papers on quarantines, lockdowns, and social distancing matters, which belonged to the procedures of the policy. In contrast with GT, the CTC method grouped the noun phrases that were associated with the same aspects, thus making the results more interpretable.

**Table 1.** Identified CTC clusters for containment and closure (CC)

CTC cluster ID	Noun phrases	Rank scores
CC1	“support mental health”, “physical mental”, “physical health”, “mental health”, “impact physical mental health”, “mental health social”, “impact physical”	4,609
CC2	“lockdown measure”, “implementation lockdown”, “northern”, “milan”, “lockdown”, “impact lockdown”, “impact lockdown measure”, “lombardy”, “northern italy”	4,488
CC3	“lockdown”, “self isolation”, “isolation”, “quarantine”, “isolation home”	2,608
CC4	“slowing”, “stopping”, “spread covid”, “public policy”, “slow spread”, “public health policy”, “spread”, “policy”, “health policy”	2,485
CC5	“social distancing”, “decision maker”, “distancing”, “social distancing isolation”, “stricter”, “distancing lockdown”, “social distancing lockdown”, “isolation social distancing”	2,464
CC6	“severe symptom”, “associated symptom”, “symptom anxiety depression”, “symptom”, “symptom pandemic”, “symptom anxiety”, “symptom depression anxiety”, “symptom depression”, “symptom time”	2,289
CC7	“student population”, “mental health student”, “student”, “college students”, “impact student”, “college student”, “health student”, “student reported”, “student mental health”	2,234

By analyzing the patterns in the occurrence of phrases and the reported COVID cases, we were able to find out how researchers responded to the COVID-19 pandemic. We specifically focused on the noun phrases within the 7 CTC clusters identified in the study, prioritizing phrases with frequencies higher than the average. These occurrences, along with the square roots of the reported COVID cases, are visually represented as a heat map in Fig. 3B. In this representation, cooler colors (i.e., green) indicate higher values, while warmer colors (i.e., red) indicate lower values. The time period considered for this analysis was from 2/1/2020 to 5/1/2020. Over these 4 months, the number of confirmed COVID cases steadily increased, with a significant surge from March to April. According to the WHO COVID-19 dashboard^5^, March 2020 witnessed a substantial rise in infections, leading to approximately half of the global population being subjected to some form of lockdown in April 2020, in an attempt to mitigate and halt the spread of the virus. This led to a relatively lower increment in the number of reported cases in May than in April, and that trend agreed with the research findings that lockdown at the early stage of the pandemic could effectively slow down or even stop the spread of the virus [19]. Next, we examined the occurrences of the selected phrases. Figure 3B shows that the occurrence of the word “spread” had a very sharp increase in March, indicating that the fast transmission nature of COVID-19 captured the attention of researchers. After a month (i.e., April), in addition to the word “spread”, the words “symptom” and “isolation” had large increases. It was natural for researchers to extend their scope of study to the isolation arrangements and their impact, because isolation was one of the very first arrangements to help protect the public. The isolation practice was to separate the infected people or potentially infected people from those who were not sick, and in April, many countries implemented lockdown policies [20]. This led to major increases in the occurrences of many related noun phrases in May, including “lockdown”, “mental health”, “policy”, and “social distancing”, each of which had a much higher occurrence in May than in April, thus indicating that researchers had extended their focus to the issues of lockdown policy, social distancing, and mental health at that point in time.

Last, it is noteworthy that the expression “mental health” appeared at the very beginning, with many research works on the topic published in May 2020 [21–23]. Figure 3A indicates that the researchers in the RAs investigated this issue much earlier than the public searching GT did, and that they foresaw mental health to be a major problem of social distancing and lockdown policy. Furthermore, we found a strong association between the expressions “mental health” and “student”. Next, we compared the Pearson correlation between the occurrences of these 2 words in the entire study period (i.e., 2/1/2020 to 8/31/2022). As there are a total of 31 months, the correlation is computed based on the number of cases and the word counts of the key phrases of these 31 months. The correlation was 0.98 and the correlation map is shown in Fig. 3C. This implies that when researchers discussed “mental health”, it was likely in regard to “students”. We found that the mental health of students was indeed one of the key scopes among the research studies. This correlation aligns with the findings of the Global Burden of Disease study, which indicates that the COVID-19 pandemic has an impact on the mental well-being of young individuals. Furthermore, the study highlights that young people are particularly vulnerable to experiencing suicidal tendencies and engaging in self-harming behaviors at a disproportionate rate [21]. In Fig. 3C, the strong correlation between the keywords and the number of confirmed cases may provide hints for predicting pandemics in the future.

In summary, the RAs included the main keywords for containment and closure much earlier than GT did, and the CTC method was able to reveal the key themes of the abstracts in each period. The trends of the occurrences of the noun phrases indicate that the researchers followed the COVID status closely and studied the major issues of the lockdown policies.

### Vaccine

**Fig. 4.**
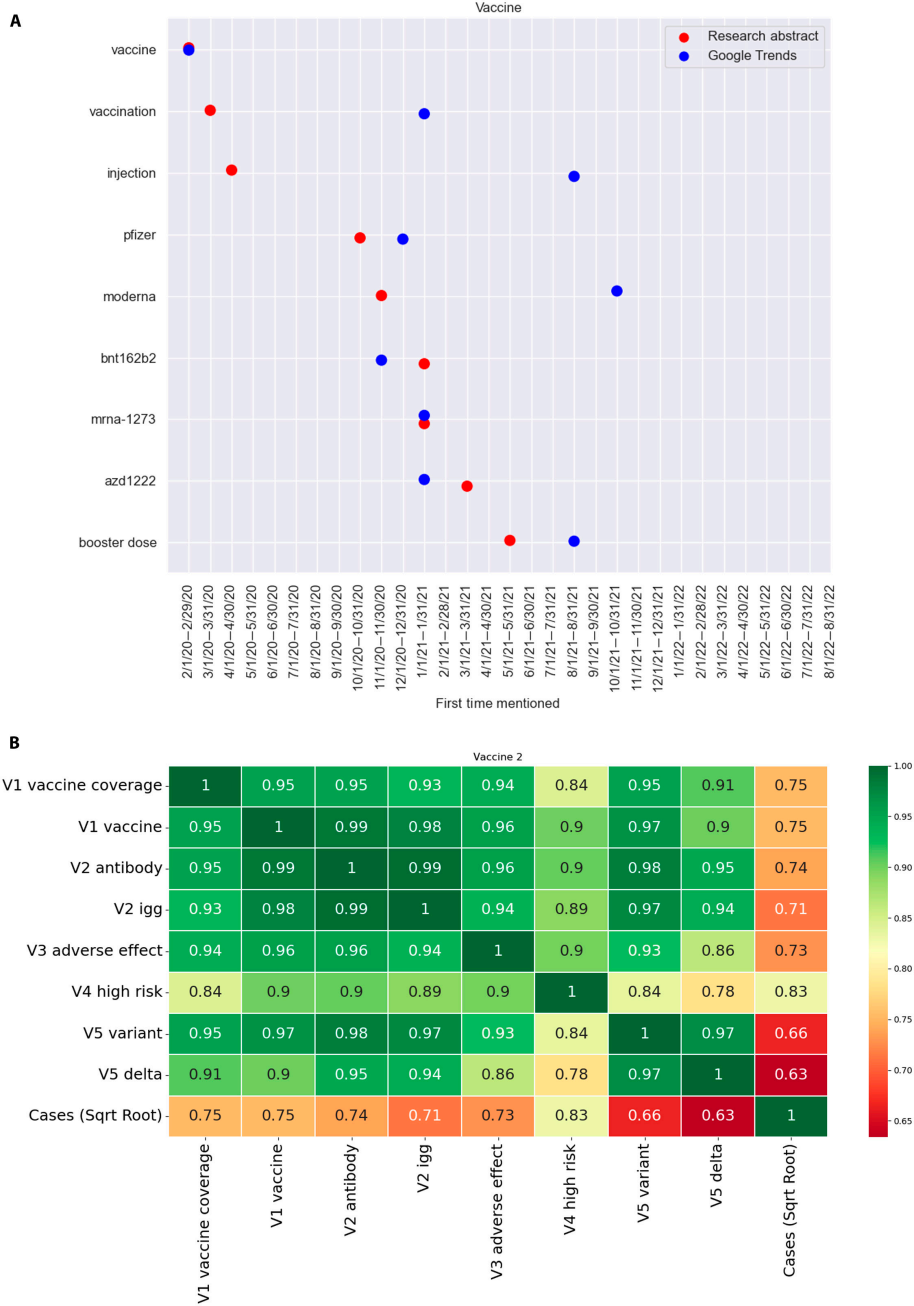
Analysis for the keywords/phrases under the category of Vaccine (V). (A) Time plot of the keywords/phrases. (B) Correlations among selected keywords/phrases and the number of reported COVID cases.

We next reviewed the response times of the GT keyword “vaccine” in GT and in the RAs. The keywords and the response times in GT and the RAs are shown in Fig. 4A. The RAs first contained most of the keywords associated with vaccines earlier than GT did. The 5th percentiles of the bootstrapping mean and median differences in the number of months between mentions of keywords for this category were 1.0 and 0.0, respectively, and the 95th percentiles were 9 and 10, respectively. In other words, the RA articles discussed these issues from 1 to 9 months (in terms of mean) and 0 to 10 months (in terms of median) earlier than the GT searches did. This also indicates that the researchers generally studied different aspects of the vaccine topic earlier than the GT public did. The figure shows that the GT searches included the keywords “vaccine”, “azd1222”, “bnt162b2”, and “mrna-1273” earlier than or at the same time that the RAs did. Vaccine referred to the liquid injected into or aerosol inhaled into a human body, while “azd122”, “bnt162b2”, and “mrna-1273” were the ingredients of the COVID vaccines. The fast response times of GT imply that the public had major concerns about the ingredients of the vaccines. Indeed, studies found that the acceptance rate of the vaccines was low in many regions [24,25]. According to Hou et al. [26], the intention to accept a COVID-19 vaccination was reported by only 36.4% (571/1,568) of individuals in New York and 51.3% (738/1,440) of individuals in London. The primary reasons cited for refusing the COVID vaccine included concerns about safety, with the belief that a vaccine developed hastily may pose significant risks. Additionally, some individuals considered the vaccine unnecessary due to the relatively mild nature of COVID-19, while others expressed skepticism due to perceived inconsistencies and contradictions in the information provided to the public [27,28]. These factors led to a large number of searches using the keywords in Google. On the other hand, the researchers needed time to study the effectiveness of the vaccine ingredients, and to do so, they had to inject the vaccine into certain patients and observe their responses to the vaccine. This introduced a time lag before the researchers were able to discuss the ingredients of vaccines. In addition to these keywords, however, there were keywords that were first used earlier in the RAs than in the GT: “vaccination”, “injection”, “Pfizer”, “Moderna”, and “booster dose”. The keywords “vaccination” and “injection” were discussed 10 and 12 months earlier in RA articles than in GT searches. This is reasonable, because at the beginning of the pandemic, researchers suggested that the vaccines were one of the most effective ways to protect communities. However, owing to the public’s reluctance to accept the vaccines, people tended to wait and take the vaccines later, after some time had passed—one possible reason for the huge time lag. In addition, “Pfizer” and “Moderna” are 2 biotechnology companies that developed COVID-19 vaccines, and the RAs contained mentions of these 2 companies 2 months earlier than the GT did, likely because the universities with which many researchers were affiliated were working with these companies to develop the vaccines, making it likely that they had to discuss things earlier.

We studied the contents of the RAs by applying our CTC method to the RAs with at least one GT keyword under the vaccine category. A total of 52,070 papers were selected, and the method produced 1,194 CTC clusters. Seven of those CTC clusters are listed in Table 2, with CTC cluster identities V1 through V5. These CTC clusters cover a wide range of vaccine-related topics. The largest CTC cluster (CTC cluster V1) referred mainly to the coverage and acceptance rates of the vaccines. The keywords indicated that the research works also discussed the issues of low vaccine rate and probably of high vaccine rate, with corresponding rank scores of 12,960. In other words, the keywords appeared on average 0.24 (12,960/52,070) times in each RA. The rank of this CTC cluster was the highest of this group of clusters, thus indicating that vaccination rate was one of the major focuses of the research at the time. It seems that researchers not only focused on the development of the vaccines but also on their acceptance by the general public. The second CTC cluster (CTC cluster V2) mainly covered the detection methods for COVID-19. The diagnosis of COVID-19 often relies on nucleic acid detection, which is a commonly used technique. However, there have been numerous reports of false-negative results associated with this method. In order to enhance the accuracy of testing, researchers have explored the potential of combining nucleic acid detection with antibody detection (specifically, immunoglobulin G [IgG] and immunoglobulin M [IgM]) [29]. This second CTC cluster had a relatively high ranking. The third and fourth CTC clusters (CTC clusters V3 and V4) represented the positive or adverse effects of vaccines to high-risk groups or patients with chronic diseases, including cancer, heart disease, diabetes, and others. As we know, the adverse effects of the vaccines are one of the concerns of vaccine-hesitant individuals [30,31]—the general public, including chronically ill patients, have concerns about the side effects or even adverse effects of the vaccines [32]. However, research has shown that if they have a good understanding of vaccines, they are more willing to accept these vaccines [33]. The keywords of these 2 CTC clusters reveal that these research studies addressed the concerns of high-risk groups and chronically ill patients. The last CTC cluster listed in Table 2 refers to the vaccines’ efficacy against different variants of COVID-19. It is noteworthy that the RAs were selected with the GT keywords in the category “vaccine”. This shows that the RAs contained the keywords of V5 and at least one of the other keywords (e.g., vaccine, injection, etc.) in Fig. 4B. These research studies reported data from tests of the vaccines against different variants of COVID-19.

**Table 2.** Identified CTC clusters for vaccine (V)

CTC cluster ID	Noun phrases	Rank scores
V1	“acceptance rate”, “vaccine coverage”, “study vaccine”, “vaccine”, “rate vaccine”, “low vaccine”, “high vaccine”	12,960
V2	“antibody”, “nucleic acid”, “positive igm”, “nucleic acid test”, “igg antibody”, “detection rate”, “antibody detection”, “positive igg”	10,520
V3	“effect vaccine”, “associated vaccination”, “potential effect”, “effect vaccination”, “adverse effect”	2,522
V4	“high risk group”, “chronic disease”, “patient chronic”, “high risk”, “risk groups”	2,509
V5	“beta delta”, “variant infection”, “delta variant”, “variant”, “different variant”, “delta”, “delta variants”	2,193

Next, we compared the topics obtained by GT and the CTC clusters of the RAs. We can see that keywords such as “azd1222” and “bnt162b2”, which were mentioned earlier in GT than in the RAs, reflected the major concerns of people around the globe. Because people generally had reservations about the effectiveness of the COVID vaccines, they searched for relevant information in Google. However, the GT keywords were not as comprehensive as the topics covered by the RAs, because the researchers considered multiple different aspects of the vaccines, including acceptance rates of the vaccines, the side effects or adverse effects of the vaccines, and the vaccines’ effectiveness against different variants. Therefore, the RAs provided a more comprehensive view and covered more aspects of the vaccine topics than GT did.

We next studied the trends of associations between the keywords’ occurrences and the count of officially confirmed COVID cases. For that, from the clustering results obtained by the CTC method, we selected 8 different keywords with high occurrences. Again, we counted the occurrences of each keyword in each month and formed a time series. We then considered the Pearson correlations between any 2 time series and the square root of the number of COVID cases. Figure 4B is the heat map of the correlations of the trends in associations. Note that the correlations among the keywords were generally very high, with most of them above 0.9. The correlations between the keywords and the numbers of confirmed cases were mostly above 0.7, showing that the number of various research studies was closely associated with the COVID-19 status. The high correlations between the keywords and the count of officially confirmed cases may provide hints on the prediction of pandemics in the future. For the keywords, the highest correlations were 0.99, for “antibody” and “vaccine” and also for “antibody” and “IgG”. These associations are reasonable because vaccines work by stimulating the immune system to produce antibodies, and IgG is a type of antibody. Both IgG and IgM were used with nucleic acid detection to improve the COVID testing accuracy [29]. In addition, the key phrase “adverse effect” was highly correlated with “vaccine coverage”, “vaccine”, and “antibody”. The public demonstrated great concern about the adverse effects of the vaccines, and such a concern generally leads to a low acceptance rate for a vaccine, which, in turn, could account for the negative impact on “vaccine coverage”. The keyword “variant” had high correlations with many of the keywords, most of which were above 0.9. This reflects the reality that the researchers tested the effectiveness of the vaccines toward different variants of COVID-19. One of the COVID variants was delta, which was spread worldwide in November 2021. There were few publications—almost none—about this variant before that month. Therefore, the correlations of this keyword’s occurrences with other keywords were relatively smaller. Another observation is that the correlations between the key phrase “high risk” were relatively smaller with the other keywords—mainly approximately 0.8 to 0.9. While being part of a high-risk group is considered a contributing factor to vaccine hesitancy, research studies have indicated that the primary reason for vaccine hesitancy is concern regarding the potential adverse effects of the vaccines. If people had a good understanding of the vaccines, they were willing to take the vaccine [32]. Thus, being in a high-risk group was a factor but not a major factor, which is why the correlation was relatively small.

Next, we looked at the correlations between the keywords’ occurrences and the number of confirmed cases. Those correlations were generally above 0.7, and the corresponding keywords were “vaccine coverage,” “vaccine”, “antibody”, “igG”, and “adverse effect”. These relationships imply that researchers were publishing more relevant papers when the pandemic was getting worse. However, the correlations between the number of confirmed cases and the keywords “delta” and “variant” were relatively lower, perhaps because the delta variant appeared in a later stage of the pandemic, thus allowing only a few relevant research studies to be reported. The correlation between the keyword “high risk” and the number of confirmed cases was the highest, at 0.83, showing that when the pandemic was worsening, more research studies were being published about high-risk people. This implies that the researchers had major concerns about high-risk individuals and their vulnerability to the virus.

In summary, the RAs generally contained keywords related to of vaccine issues earlier than GT did. Although the GT searches included the vaccine ingredients (“azd1222”, “bnt162b2”, and “mrna-1273”) earlier than the RAs did, the RA entries covered more in-depth discussions and aspects of the vaccines, including the acceptance rates of the vaccines, the side effects or adverse effects of the vaccines, and also the vaccines’ effectiveness toward different variants. Moreover, the keywords’ occurrence trends were highly correlated with the number of confirmed cases. This shows that research work closely followed the COVID-19 status.

### Economic issues

No keywords obtained from the GT searches were related to economic issues, whereas economic topics were found in the RAs. This shows that economic concerns in relation to COVID-19 were not among the major concerns of public. This situation is similar to other categories, with the public only searching the internet for information on matters about which they felt the most concerned. In the vaccine category, the public showed great concern about the ingredients of the vaccines and searched for relevant information. Vaccine ingredients could have a negative impact on the human body that is even life-threatening. Based on the hierarchy of needs, individuals must satisfy their fundamental physiological needs such as food, water, and rest, as well as their safety needs, which involve security and protection, before they can pursue other needs. This could potentially clarify why no economic-related term was identified in the GT.

Next, we examined the content of the RAs under the category of economic topics. Because no such keywords were obtained from GT, we considered the keyword to be “economic” and selected the RAs that contained this keyword. We found a total of 31,938 papers, and then applied the CTC method to those papers, with a resulting 737 CTC clusters. We selected 3 of the clusters for discussion, and they are listed in Table 3. The rank scores of the first CTC cluster were 2,365. The identified keywords related to the financial situation of small enterprises, and research has demonstrated the severe impact of COVID-19 on these businesses [33]. In 2020, a survey conducted among 10,000 small business owners revealed that the coronavirus had already affected 96% of them, and 51% expressed concerns that their business would not be able to endure a 3-month economic shutdown [34].

**Table 3.** Identified CTC Clusters for economic (E)

CTC cluster ID	Noun phrases	Rank scores
E1	“figure”, “dollar”, “economic”, “substantial”, “small business”, “billion”, “firm”, “businesses”, “illustrate”, “trillion”	2,365
E2	“lower income”, “factor affecting”, “affecting”, “household income”, “income group”, “income”, “higher income”	1,096
E3	“food supply chain”, “food supply”, “food system”, “impact food”, “food safety”, “global food”	1,090

The economic factor that impacted household income was the second CTC cluster. Studies indicated that households with low income and those relying on labor income were particularly susceptible to a sudden decline in earnings, resulting in poorer food consumption during the COVID-19 pandemic compared to other groups of respondents [35].

The final CTC cluster focused on the food supply and its chain. During the peak of the pandemic, COVID-19 had adverse effects on the food supply chain, causing significant disruptions and even resulting in the near-complete loss of the food service distribution network [36]. These were major events that affected nearly everyone around the world. However, the relevant keywords did not appear in GT. From this perspective, we can see that the contents of the RAs provide a more comprehensive view of the economic issues related to the COVID-19 pandemic.

**Fig. 5.**
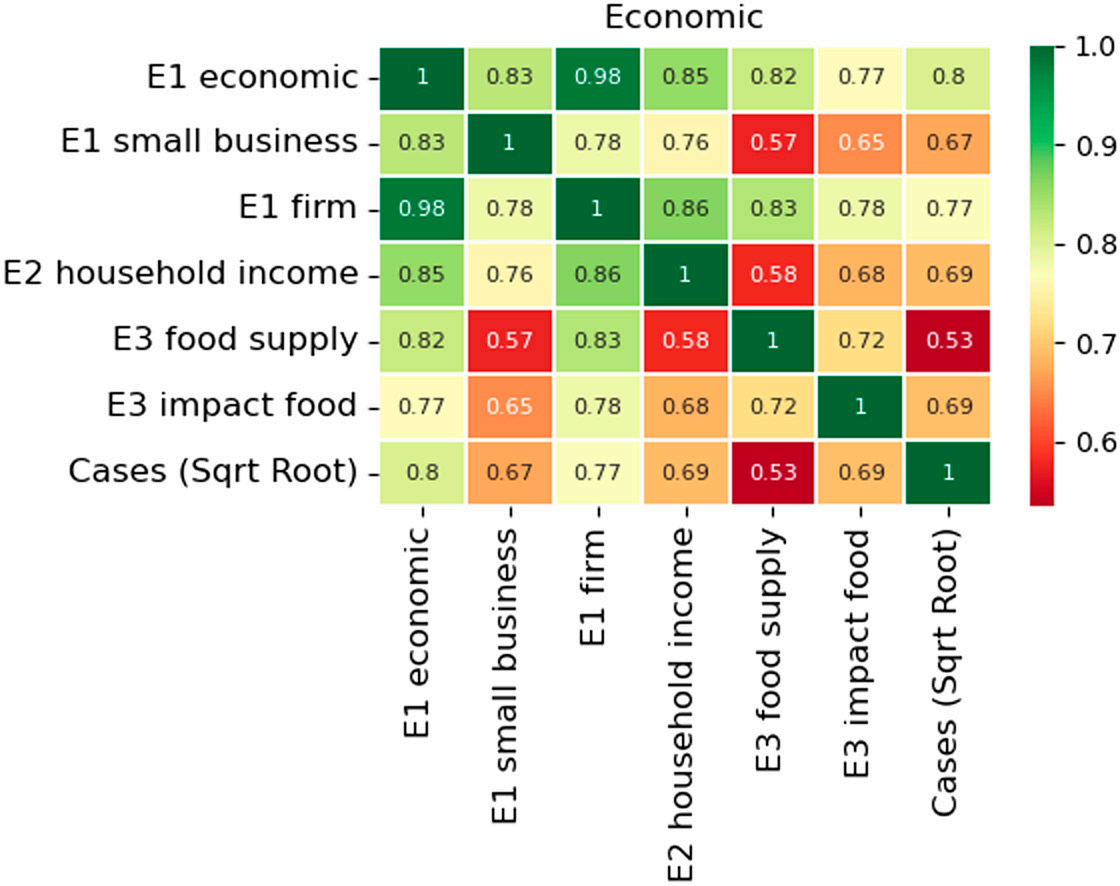
Correlations among selected keywords/phrases and the number of reported COVID cases under the category of economic.

Next, we studied the trends in the associations between the keywords’ occurrences and the number of confirmed COVID cases. We selected 6 different keywords from the CTC clusters and counted the occurrences of the keywords monthly to form a time series. We then found the Pearson correlation coefficients among the keywords and the correlation with the square root of the confirmed COVID cases. The correlations are shown as a heat map in Fig. 5. There were 2 groups of high-association clusters, the first of which was “economic”, “small business”, “firm”, and “household income”. This group revealed that the economic status of firms and small businesses had a direct impact on the household income of families. The second cluster consisted of the categories “economic,” “food supply,” and “impact food,” which were all interconnected and related to the effects of COVID-19 on the food supply chain. Therefore, by considering the word correlations, we identified the major economic issues associated with the COVID-19 pandemic.

Finally, we investigated the linkages between the frequency of keyword occurrences and the number of confirmed COVID-19 cases. The strongest correlation, with a value of 0.8, was found between the keyword “economic” and the number of confirmed cases. This correlation indicates that researchers focused more on studying economic issues when the number of cases was high. The second highest correlation, at 0.77, was observed between the keyword “firm” and the number of COVID-19 cases. The third highest correlations were found between the keywords “household income” and “impact food” with the number of COVID-19 infections. These correlations shed light on the economic aspects that researchers considered in relation to the COVID-19 situation, and it was observed that a greater number of relevant studies were published as the number of cases increased.

In summary, the RAs contained keywords for the major economic issues that were under the influence of COVID: the impact of the disease on firms, on household income, and on the food supply chain. The keywords were also highly correlated with the number of confirmed cases, implying that the researchers published more frequently when the pandemic was getting worse. However, no economic keywords were found in the GT, perhaps because the public only searched for their own major concerns. According to the life pyramid of needs, economic issues are not among the top priority life concerns.

## Discussion

The findings of this study can serve as evidence to guide decision-making processes and should benefit policymakers. Because worldwide information provides overall views of various issues around the globe, we considered the keywords obtained by GT and the contents of the RAs in the WHO COVID-19 database. It is found that GT can overlook factors such as “economic”, perhaps because the public usually searched for the matters that concerned them the most, whereas the factors considered by the researchers were more comprehensive. However, these are important pieces of information that can help policymakers to make decisions.

Although a multitude of different topic-modeling methods have been developed, many of them have several limitations that hinder their applicability for solving real-world problems. One of the most widely used topic modeling tools is latent Dirichlet allocation (LDA) [37]. First, LDA requires users to specify the number of topics, but this is usually not known in advance. Despite the existence of various methods suggested for estimating the ideal number of topics, these approaches often rely on a trial-and-error process that can be excessively time-consuming [38,39]. The core idea of those methods is to evaluate the LDA under different numbers of topics using a predefined objective function—that is, LDA must be applied at least once for each proposed number of topics. The optimal number of topics is then the one that can optimize the objective function. Consequently, if the optimal number of topics is small, this may not be a problem, but for a large amount of textual data, the optimal number of topics can be huge. If the optimal number of topics is 1,000, LDA has to be applied 1,000 times—which can be very time-consuming. Additionally, the LDA technique initiates topics randomly, and different random seeds can yield varying outcomes. Therefore, to achieve the best performance and determine the optimal number of topics using LDA, it is necessary to repeatedly apply LDA with the same number of topics. This iterative process can also be quite time-consuming. Furthermore, the topics generated by LDA can be challenging to interpret as they consist of words from different documents. Some researchers have compared LDA with simple co-occurrence analysis and found that LDA can uncover latent semantic connections between words, even if they do not actually occur together in any document [40]. If the words from the same topics are from different documents, the meaning of the topic can be very diverse.

To address the limitations of the existing topic modeling tools, therefore, we have proposed a new topic modeling method—the CTC method. The key idea of the proposed method is to extract the noun phrases that frequently appear together in the same abstracts. As the phrases appear frequently together, they should be discussing the same issues. They should be the major themes of the abstracts as well. This approach is very different from many existing topic modeling tools. Beyond that, we introduced a distance threshold to group the phrases together. If the similarity between topics was larger than the distance threshold, they were to be further broken down into additional topics. This automatically selected the number of topics, so that the users did not have to specify this parameter. In fact, we used the same distance threshold in all of the experiments in this paper, and our results indicated that the threshold was robust.

Next, we compared the performance of LDA and our method. We considered the RAs under the category of containment and closure, and we applied LDA to the 93,378 papers and assumed that there were a total of 2,329 topics (refer to the section on “Containment and closure” for more details). The LDA required 14,145 s (nearly 4 h) to generate its results, while the proposed method used only 109 s. The difference in computational times was so large because LDA adopts an iterative approach to form the topic clusters [37], and the iterative approach has to repeatedly compute some statistics such as weighted means many times. This can be a huge computational burden to many computers. The proposed method, in contrast, used a distance threshold to group the phrases. The threshold directly broke down the topics into many subtopics, and no iterative procedure was used. This approach not only reduces computational time significantly but also allows for the estimation of the ideal number of topics.

Next, we compared the results obtained by LDA and the results from our topic coherent clustering method. The top 3 topics extracted by LDA are shown in Table 4. Because LDA adopts single words as the data input, it is hard to interpret the actual meaning of the topics; moreover, the single words seem to be from different documents. For example, “harvest” and “biometrics” should refer to 2 different things. The results of our method were clusters CC1 to CC7 in Table 1. Because noun phrases were used, each element was easier to understand. Furthermore, the phrases in the same clusters were likely to have been drawn from the same abstracts, and that knowledge can help human users interpret the meanings of the clusters.

**Table 4.** Results of applying LDA to the research abstracts (RAs) in the category of containment and closure

Topic	Words
Topic 1	harvest, biometrics, linguist, mcarthur, renowned, sanskrit, scots, agro, dweller, farsi
Topic 2	care, case, age, year, report, clinic, reported, scots, dweller, agro
Topic 3	study, health, data, covid, social, time, level, symptom, public, individual

We compared the proposed CTC method with a recent advanced deep learning-based topic modeling, BERTopic [41]. BERTopic has 3 main parts. First, it embeds documents using sentence-BERT (Bidirectional Encoder Representations from Transformers) [42], which converts sentences and paragraphs to dense vector representations using pre-trained language models. It then uses HDBSCAN (Hierarchical Density-Based Spatial Clustering of Applications with Noise) [43] to cluster the relevant documents together. Finally, it generates topic representatives for each cluster by extracting the word representatives from the clustered documents using a modified TF-IDF (Term Frequency–Inverse Document Frequency) [44] measure. We use the default setting of BERTopic except that we set the parameter n_gram_range to be (1,3) to output word/phases. The top 3 topics obtained by BERTopic are shown in Table 5. Compared with LDA, the results are more interpretable. However, the results are different from the proposed CTC.

**Table 5.** Results of applying BERTopic to the research abstracts (RAs) in the category of containment and closure

Topic	Words
Topic 1	students, learning, education, online, teachers, university, student, educational, online learning
Topic 2	loneliness, older, older adults, adults, social, social isolation, loneliness and, of loneliness, and loneliness
Topic 3	physical activity, physical, activity, pa, exercise, sedentary, of physical, of physical activity, physical activity and, activity and

We compare the contents obtained by BERTopic and the proposed CTC by applying the co-occurrence analysis to the RAs. Word co-occurrence analysis involves tallying the occurrences of 2 words appearing in the same documents. If the word count is larger, the words have higher correlation and better represent the contents of the RAs. Table 6 shows the co-occurrence counts. The co-occurrence counts of the proposed CTC are generally larger than BERTopic. This implies that the RAs of this category should be more about support and impact of mental health and also implementation, impact, and measure of lockdown rather than student’s learning and adult loneliness. In Topic 1 of BERTopic, the keywords seem to be about students’ learning and the word counts are large. However, after reviewing the RAs, there are over 1,200 abstracts with keywords “education” and “online” that are not related to student’s learning. These 2 keywords are about online survey/questionnaire. The most frequently asked questions are “education” level and “student” status. For the proposed CTC, there is also a cluster about students (CC7). It is related to mental health/health. After reviewing the RAs, the abstracts are mainly about the mental health/physical health/health of students. One possible reason why BERTopic cannot discard the irrelevant cases may be owing to the use of unigram (single word) to trigram (3 conservative words) as data input. As “education”, “online” and “student” may have high correlation in the sentence-BERT space (first step of BERTopic), they can be grouped together and formed a cluster. The sentence-BERT space is a pre-trained language model, which was trained based on the information given by Wikipedia and huge volume of books. However, for the proposed CTC, it recognizes “education level” and “online survey” as noun phrases. They do not frequently appear with “students”, and thus, these noun phrases are filtered out.

**Table 6.** Co-occurrence counts of term 1 and term 2 of the RAs in the category of containment and closure. The numbers in parentheses show the number of co-occurrences of the 2 words.

	Term 1	Term 2				
BERTopic						
Topic 1	student	learning (2,774)	education (3,295)	online (3,342)	teacher (1,041)	online learning (680)
	learning	education (2,588)	online (2,419)	teacher (941)		
Topic 2	loneliness	adult (671)	social (1,464)	social isolation (641)		
Topic 3	physical	activity (3,300)	exercise (1,052)	sedentary (416)		
CTC						
CC1	mental health	social (7,606)	support (6,031)	physical (4,542)	impact (8,618)	
CC2	lockdown	measure (8,680)	implement (3,827)	impact (10,070)		
CC3	quarantine	lockdown (1,581)	isolation (1,963)			
CC7	student	mental health (3,021)	health (4,104)	impact (2,528)		

The above results show that the proposed CTC can extract more accurate content than BERTopic. One possible reason why BERTopic did not perform as good as the proposed CTC method is that BERTopic uses a modified TF-IDF measure to find the word representatives of each cluster. The measure gives a large weight to indicate the importance of a word of a cluster if this word is relatively rare among all clusters and, otherwise, a small weight to a word of a cluster if this word is relatively common. This procedure should extract words for each cluster that are as different as words from other clusters. However, this difference searching procedure may not find words that appear frequently among all the documents. In contrast with BERTopic, the proposed CTC finds a set of words that frequently appear together. These can better represent the content of the huge number of RAs.

## Conclusion

We proposed a new research framework to analyze 2 worldwide bodies of information about COVID-19, which are the keywords of GT and RAs downloaded from WHO research database and discussed their response times to different COVID issues. We also proposed a new text-mining method, CTC method, that can group key phrases that frequently appear in the same abstracts. This can effectively find the major themes of the RAs. In our analysis, it is found that the RAs not only addressed many COVID-19 topics prior to GT, but they also offered more comprehensive and detailed coverage. This can assist policymakers in identifying key COVID-19 concerns and taking timely action.

### Ethical approval

There are no human subjects in this article and informed consent is not applicable.

## Notes

1. Kaggle. COVID-19 Open Research Dataset Challenge (CORD-19). 2022. [accessed 23 March 2023] https://www.kaggle.com/datasets/allen-institute-for-ai/CORD-19-research-challenge
2. WHO. WHO COVID-19 research database: Global research on coronavirus disease. 2023 [accessed 23 March 2023] https://search.bvsalud.org/global-literature-on-novel-coronavirus-2019-ncov/
3. Zenodo. spaCy: Industrial-strength Natural Language Processing in Python. 2023. [accessed 10 March 2023] https://doi.org/10.5281/zenodo.1212303
4. Blavatnik School of Government. COVID-19 Government Response Tracker. 2021. [accessed 23 March 2023] https://www.bsg.ox.ac.uk/research/covid-19-government-response-tracker
5. WHO. WHO coronavirus (COVID-19) dashboard. 2023. [accessed 23 March 2023] https://covid19.who.int/

## Data Availability

This study used the keywords downloaded from Google Trends and the research abstracts downloaded from WHO website. They are free and publicly available.

## Supplementary Materials

Supplementary 1 Supplemental materials for this article are available online.
