# Supplementary material for: Do Scholars Respond Faster Than Google Trends in Discussing COVID-19 Issues? An Approach to Textual Big Data
Source: Health Data Sci. 2024 Feb 26;4:0116. doi: 10.34133/hds.0116 (PMC10895931; doi:10.34133/hds.0116)
Supplement: Supplementary 1 — Supplemental materials for this article are available online. [file hds.0116.f1.docx]

Contents

[Appendix M1 - Grouping the GT Keywords 2](#_Toc152941577)

[Appendix M2 – Comparing Response Time of GT and RAs Using Bootstrapping Statistics 3](#_Toc152941578)

[Appendix M3 - Health System 4](#_Toc152941579)

[Appendix M4 - Country 9](#_Toc152941580)

[Appendix M5 - Others 11](#_Toc152941581)

# Appendix M1 - Grouping the GT Keywords

We used the hierarchical clustering method with a complete link to group the keywords downloaded from GT. We counted the number of co-occurrences of the GT keywords among all of the RA entries and denoted them as ${CO}_{ij}$, where $i$ and $j$ were indices representing the $i$th and $j$th GT keywords. The distance matrix was then computed by the following formula:

$D_{ij}=1-CO_{ij}/\max_{i,j} CO_{ij}$.

The number of clusters was determined by locating the largest vertical difference between nodes in the dendrogram. A total of 50 clusters were found.

# Appendix M2 – Comparing Response Time of GT and RAs Using Bootstrapping Statistics

We constructed 95% confidence intervals using bootstrapping to compare the response times between GT and RAs in each category. We sampled the first response time of the category with replacement with a sample size equal to the number of keywords. If there are 10 keywords, the sample size is 10. We then computed the mean and median of these samples. We repeated this entire procedure 10,000 times. The 5^th^ percentile of the means and the medians were the lower limit of the confidence interval, whereas the 95^th^ percentile was the upper limit. We can illustrate this method by the category of containment and closure. Figure M2.1 (also shown in Figure 3(a) in the manuscript) shows the first times that these seven keywords of this category were mentioned in both GT and the RAs. We first obtained the time difference between the date of the first mention of these seven keywords in GT and the RAs, and then we found the bootstrapping means of these seven time differences 10,000 times, and reported their 5^th^ percentile and the 95^th^ percentile.

| 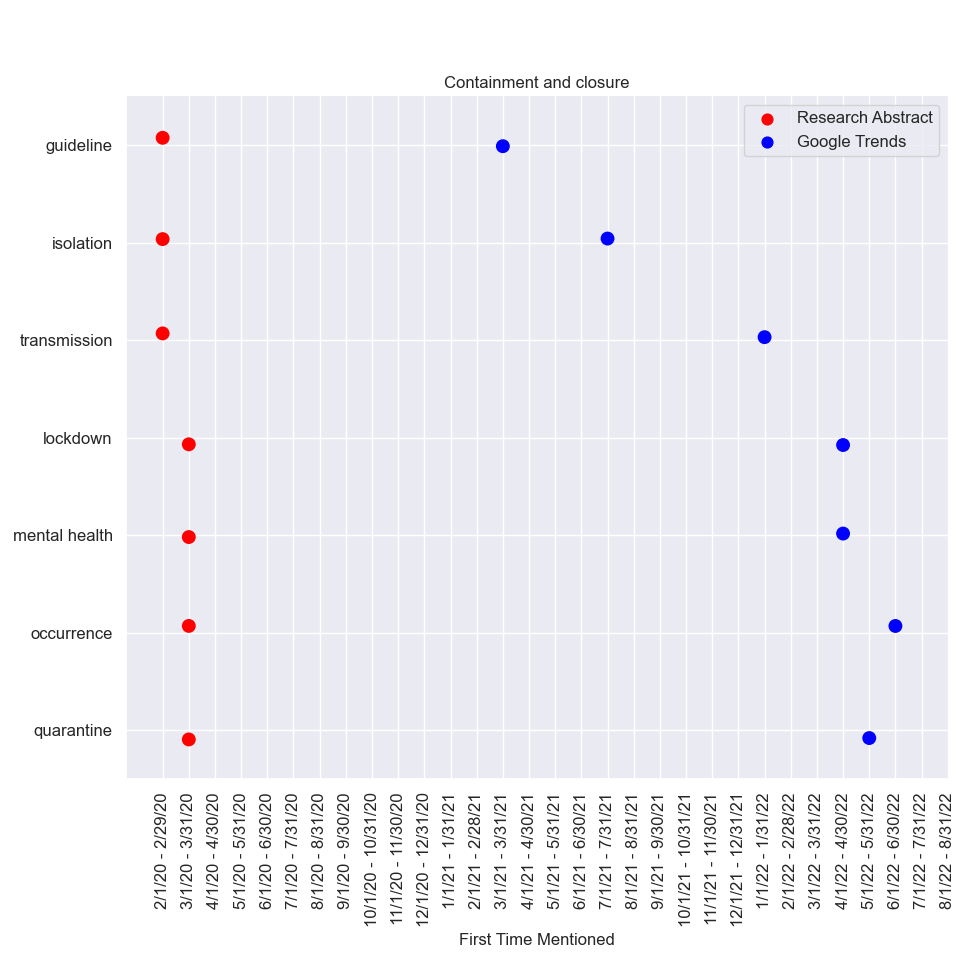 |
| --- |
| Figure M2.1 Containment and closure |

# Appendix M3 - Health System

The times when the GT keywords in the category “health system” were first mentioned in GT and the RAs are shown in Figure M3.1(a) (in this appendix). The figure shows that the RAs contained mentions of the keywords earlier than GT did in many cases. For this category, the 5th percentile of the bootstrapping mean and median differences, in numbers of months, were $3.83$ and $7,$ respectively. In other words, the RAs included discussions of these issues an average of 3.83 months and a median of 7 months earlier than GT did, and both differences were significantly greater than zero. This also indicates that the researchers generally studied different aspects of the health system earlier than the GT searchers did.

Next, we pondered some possible reasons why mentions of certain keywords relating to health systems appeared earlier in the RAs than in GT, and why other keywords appeared in the RAs later than in GT. We grouped the keywords into three classes: (i) COVID detection and testing; (ii) virus mutation; and (iii) infection and mortality. For the category (i) COVID detection and testing, the RAs contained mentions of the following keywords earlier than GT did: “serology” (1 month earlier), “polymerase chain reaction” (2 months earlier), “infection” (6 months earlier), and “reverse transcription polymerase chain reaction” (13 months earlier). However, the first RA mentions of “rapid antigen test” appeared five months later than in GT. The key is likely that these two sets of keywords refer to different concerns for testing and detection of COVID. At the early stage of the pandemic, researchers proposed accurate tests to detect the virus: serology tests, polymerase chain reaction (PCR) tests, reverse transcription polymerase chain reaction (RT-PCR) tests, and so on. These are the keywords that the RAs mentioned earlier than GT did. However, owing to the large-scale infection rate and timely results for accurate testing, rapid antigen detection tests (RADTs) were also widely used. The difference in the timing of when the two databases mentioned the terms shows how the concerns of the researchers differed from those of the worldwide public. From the researchers’ point of view, the priority was the accuracy of the test, whereas mass screening was the major concern of the worldwide public, which is why the RAs mentioned mass screening topics (i.e., “rapid antigen test”) later than the GT did. In regard to the topic of (ii) virus mutation, the RAs contained the keyword “mutation” 10 months earlier and “variant” 14 months earlier than the GT did, whereas discussions of the keyword “omicron” appeared one month later in the RAs than in GT. The figure shows that the keywords “mutation” and “variant” were first discussed by researchers in the initial few months of the pandemic, which shows that researchers began studying the variants of the virus at the very beginning of the pandemic. This is likely a common practice of biological researchers––the mutation and evolution of viruses is common and captures the attention of researchers at the beginning of a pandemic. However, GT had a first mention of “omicron” one month earlier than the RAs did, perhaps owing to the high transmission rate of this variant. Researchers found that the transmission rate of Omicron was much higher than Delta’s, which was much higher than Alpha’s (Liu and Rocklöv, 2022). The high transmission nature of the virus may have made the worldwide public feel very concerned about the virus and may have led to a large volume of search activity by the public for relevant information via Google in a short period of time. For the category (iii) Infection and mortality, the first mentions of the keywords “airborne transmission” and of “sore throat” occurred in the RAs 3 months earlier than in GT, whereas the first mentions of the keywords “statistic” and “mortality rate” occurred one month later in the RAs than in GT. This indicates that the public had a greater concern about death from the virus than they did about the methods of its transmission. As of February 28, 2020, the cumulative number of reported deaths in China was 2,858. Also, according to a timeline of the COVID pandemic (Taylor, 2023), the first deaths in other countries, including the Philippines, France, and the U.S.A. were reported at about that time. These reported deaths may have led to a high search rate in GT.

Next, we considered possible reasons why the RAs had the first mention of “mortality rate” later than GT did. We reviewed the RAs, looking for this keyword in articles that were published in February 2020, and found that one of the themes of the research studies was the infection history of the virus, such as the history of exposure to the Hunan seafood market. Thus it seems that researchers focused on the cause of the deaths rather than on the number of deaths caused by the virus.

We then studied the contents of the RAs by applying the proposed CTC method to the abstracts with at least one of our keywords, as is shown in Figure M3.1(a) (in this appendix). A total of 267,878 papers were selected, and the method generated 6,689 CTC clusters. As we had in the analysis of containment and closure, we sorted the CTC clusters for health system according to their proportions of occurrences in the abstracts and denoted them as rank scores. We then selected eight of the CTC clusters, and their corresponding keywords are listed in Table M3.1 (in this appendix) with CTC cluster IDs HS1 through HS8. The first two CTC clusters (HS1 and HS2) were about testing for and infection with COVID, with the highest corresponding rank scores at 42,293 and 32,715, respectively. The third CTC cluster was mainly about the management and support of hospitals. According to the RA results, those keywords referred to the study of the virus in the hospitals and to the overburdening of hospitals during the peak time of the pandemic. In the fourth CTC cluster, the keywords referred to the research studies over different age groups. Because the RAs contained any one of the keywords shown in Figure M3.1(a), those keywords referred to studies of COVID antibodies and infection in different age groups. We verified these connections by reviewing several of the RA articles. The CTC clusters HS5 and HS6 were about the antibodies and vaccines, and CTC cluster HS7 was about food service. According to the RA abstracts, the articles with those keywords were related to the food supply chain and shortages of food. The final CTC cluster was focused on mental health issues. In contrast with the GT search results, the keywords of the RAs provided a wide spectrum of topics, including overburdened hospitals, food supply chain issues, and mental health problems, all of which were important issues, especially at the peak of the pandemic. However, those topics were not present in the GT searches, perhaps because GT presents the keywords that are frequently searched by the public and therefore represent the matters about which the public is most concerned. In the GT results, SARS-CoV-2 detection, mutation, and infection represented topics that were relatively more life-threatening than other issues and thus may have driven the public to search relevant information in Google. In contrast, researchers tended to have a more in-depth view of COVID-related problems. From the policy point of view, the RAs represented a larger number of potential issues of COVID than the GT results did, and some of them also provided possible solutions. The topics of infection in the HS2 cluster, food service in the HS6 cluster, and mental health in the HS8 cluster are issues associated with COVID-19, whereas the topics of tests in the HS1 cluster, and antibodies and vaccines in the HS5 and HS6 clusters, are feasible solutions to several issues.

We also studied the trends in correlations between the keywords’ occurrences in the RAs and the number of confirmed COVID cases. These correlations presented the response of the researchers to the pandemic. We selected the keywords obtained by the proposed CTC method that had high occurrences throughout the time periods (i.e. 2/1/2020 to 8/31/2022): “infection,” “hospital,” “antibody,” “vaccine,” and “mental.” The trends of the correlations are shown in Figure M3.1(b), with the trend of each keyword having been obtained by counting the respective occurrences of that keyword each month. The trend of the confirmed COVID cases was obtained by taking the square root of the total number of confirmed cases each month. The correlations between a keyword and the number of COVID cases at the time were then obtained by finding the Pearson correlation coefficients for any of the two trends. The figure shows that the correlations between the keywords and the square roots of the number of confirmed cases was at least 0.77, thus demonstrating that the overall patterns between the keywords’ occurrences and the confirmed cases were similar. The five selected keywords all represented health-related issues, and the high correlations between the keywords and the confirmed cases reveal that when the situation of the pandemic was getting worse, the researchers generally published more articles to address the problems, and when the pandemic was less serious, the researchers were more likely to shift their focus onto other issues. In addition, the high correlation between the keywords and the number of confirmed cases may provide hints for predicting pandemics in the future. The highest correlation was 0.8––the correlation between the keyword “mental” and the number of confirmed COVID cases. We randomly reviewed several RA articles with the keyword “mental” and found that those research works generally discussed the mental health issues or even mental problems of the public. The researchers also discussed factors influencing the mental health of public, including fear of COVID-19 infection, unemployment, shortage of food supply, and so on. Furthermore, the correlations between any pair of keywords’ trends were high, ranging from 0.88 to 0.99. meaning that the overall occurrence trends of these keywords were also very similar to each other. The correlations between the keywords “infection,” “hospital,” “antibody,” and “mental” were very high––between 0.98 and 0.99––showing that when the researchers discussed the issues relating to infection, they were related to “antibody,” “hospital,” and “mental” as well. As discussed above, research has shown that COVID-19 infection can have a major impact on mental health.

Next, we examined the association between the keyword “infection” and the two keywords “antibody” and “hospital,” by reviewing the relevant RA abstracts. The abstracts with these two keywords were from studies of infected patients in hospitals. Researchers examined the health status and the levels of antibodies of the hospitalized patients and reported their recovery time, discharge dates, and so on. In addition, during the peak time of the pandemic, the number of infected cases was huge, and many hospitals received massive numbers of patients and were overwhelmed.

Researchers also studied the support and management of hospitals, topics that were represented by the keywords of CTC cluster HS3 in Table M3.1. One observation was that the correlation between “infection” and “antibody” was higher than that of “infection” and “vaccine”––a reasonable relationship because “antibody” can refer to both COVID infection testing and COVID vaccines. During the peak time of the pandemic, there was a great need for COVID infection testing and detection, with many people wanting to check whether they were infected. However, the general public had reservations about the effectiveness of the vaccines. That was one reason why the keyword “infection” had a higher correlation with “antibody” than it did with “vaccine.” The above word associations show that the correlations can reveal the major COVID status and their relevant issues.

In summary, in general the RA abstracts contained this study’s keywords relating to the health system earlier than GT searches did. The CTC approach was again able to reveal the key themes of the RAs. The keywords’ associations can provide useful information to policymakers about the major issues of infection and of the health care system. Moreover, the keywords’ occurrence trends were highly correlated with the number of confirmed cases, showing that the research reports followed the COVID status closely.

| 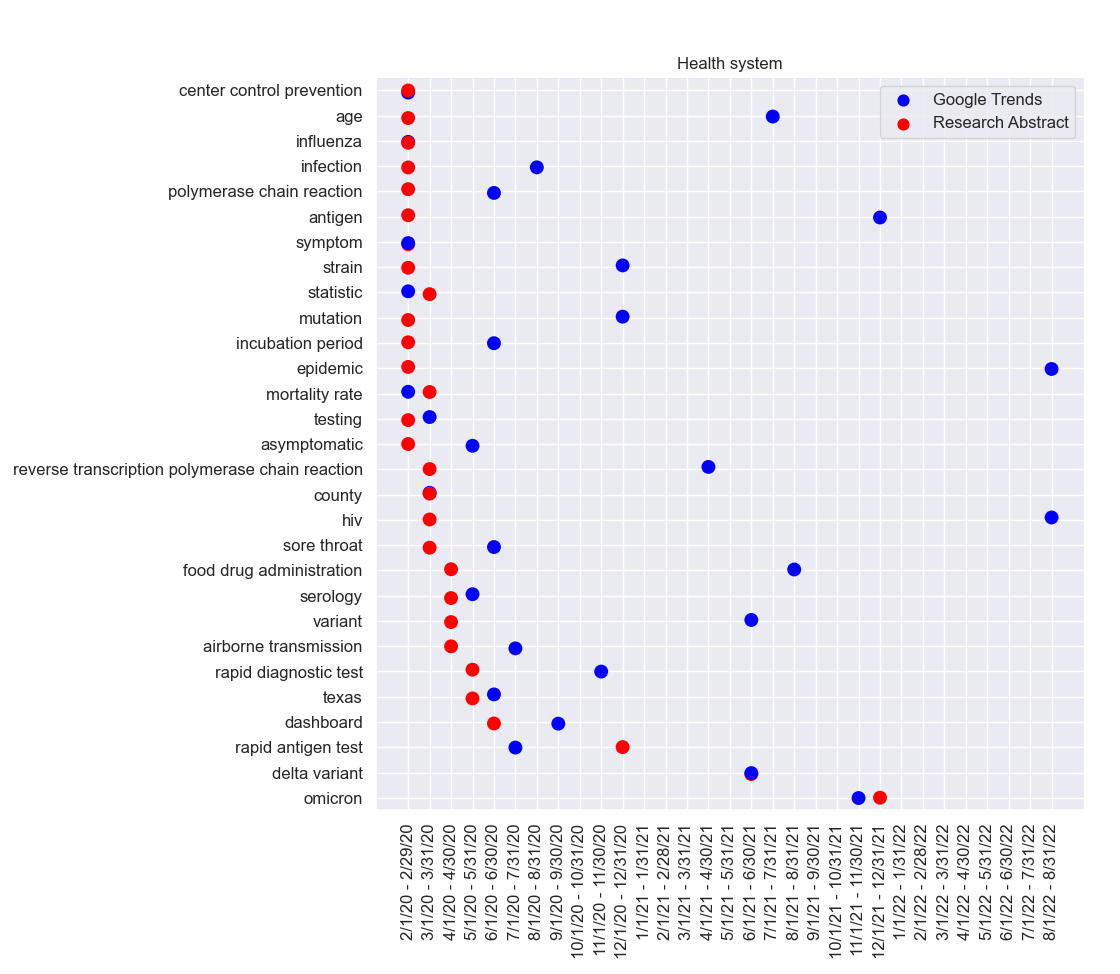 |
| --- |
| 1. Time plot of the keywords/phrases |
| 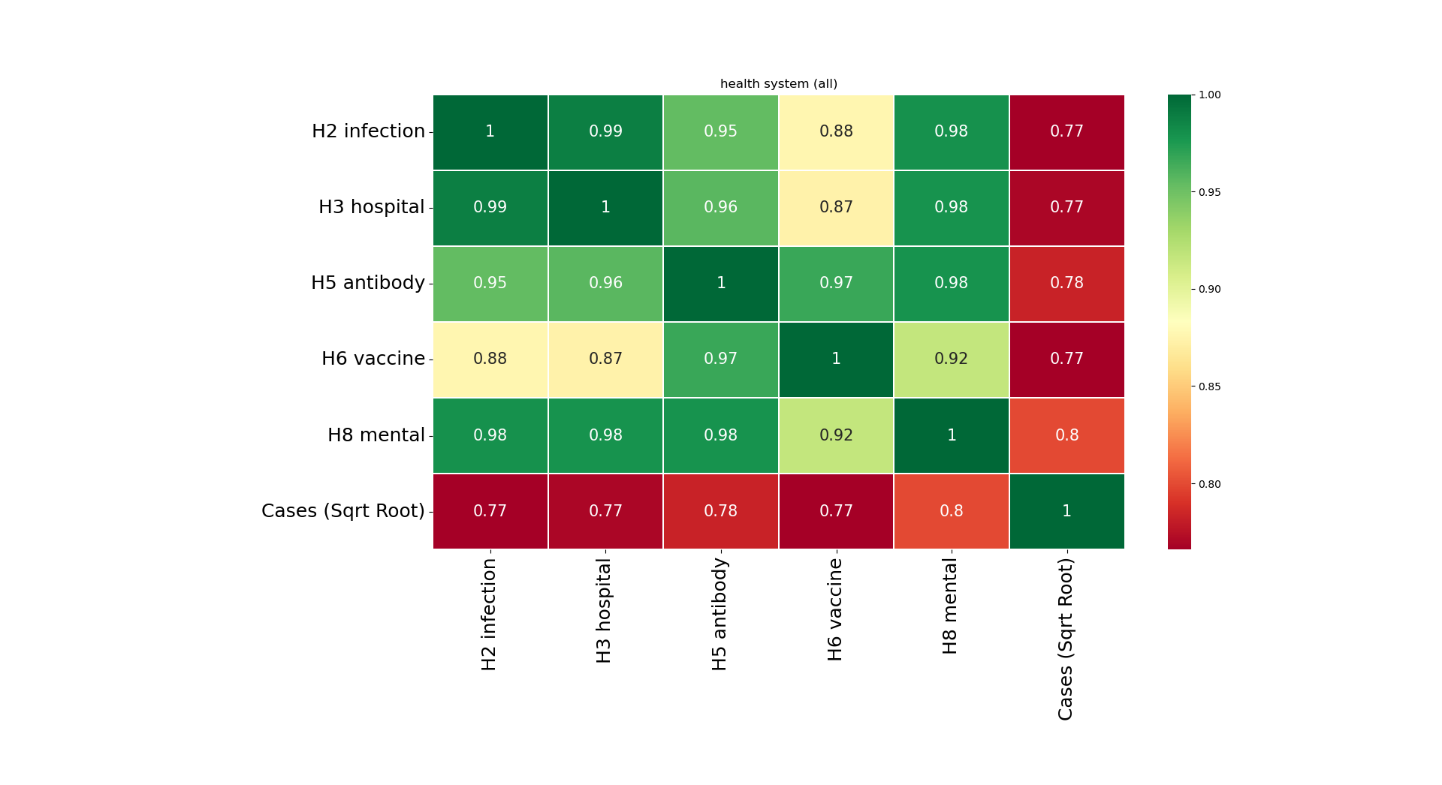 |
| b. Correlations among selected keywords/phrases and the number of reported COVID cases. |
| Figure M3.1. Analysis for the keywords/phrases under the category of Health System (HS). |

| **CTC Cluster ID** | **Noun Phrases** | **Rank Scores** |
| --- | --- | --- |
| HS1 | “covid test”, “covid”, “covid testing”, “prior covid” | 42,293 |
| HS2 | “study infection”, “data infection”, “infection” | 32,715 |
| HS3 | “hospital management”, “support hospital”, “hospital” | 18,906 |
| HS4 | “younger adult”, “middle aged”, “design research methods”, “aged adult”, “younger older”, “age group” | 17,594 |
| HS5 | “antibody”, “pcr antibody”, “antibody tests” | 10,942 |
| HS6 | “existing vaccine”, “vaccine strain”, “attenuated”, “virulent”, “vaccine”, “live attenuated vaccine”, “virulence”, “attenuated vaccine” | 8,258 |
| HS7 | “food service”, “service industry”, “service”, “service sector” | 7,181 |
| HS8 | “mental”, “mental health condition”, “mental health issue”, “mental health diagnosis” | 7,170 |
| Table M3.1. Identified CTC Clusters for Health System (HS). | | |

# Appendix M4 - Country

In addition to the four categories suggested by OxCGRT, we also found keywords obtained by the GT that related to countries, thus indicating major concerns of the regional netizens. The response times of these keywords are presented in Figure M4.1, which shows that the RAs contained more than half of the keywords earlier than GT did, and we observed that one of those keywords had a huge difference in the response times––the RAs contained the keyword “Taiwan” 25 months earlier than the GT did. This may have been due to the implementation of various measures by Taiwan that were effective in slowing down or stopping the spread of the virus (Zhao et al., 2020). However, according to the figures from WHO, the number of new confirmed COVID cases in Taiwan exceeded 66,000 in May 2022, and that may have led Taiwan’s netizens to search for relevant COVID information. This idea is supported by observing in Figure M4.1 that the keyword “Taiwan” was mentioned for the first time in GT in May 2022.

Next, we considered the overall response times of the country category keywords in the GT and RAs. The 5th percentile of the bootstrapping differences in the mean and median numbers of months between first time the keywords were mentioned in the RAs and in GT under the country category were $-0.94$ and $-2,$ respectively, and the 95th percentiles were 4 and 2, respectively. In other words, the RAs included mentions of these issues at the largest average lag of 0.94 months (in terms of mean) and of 2 months (in terms of median) before their mention in the GT. However, because the 5th percentile of these two measures were negative, the initial mentions of country keywords in the RAs were not significantly earlier than those in GT. We also observed in the figure that the keywords of certain of the Canadian provinces were usually mentioned first in GT: “alberta,” “british columbia,” “manitoba,” “new brunswick,” “nova scotia,” “ontario,” and “saskatchewan.” We grouped these keywords as the Canada group, and the rest as the non-Canada group. The 5th percentile of the bootstrapping mean and median differences in the number of months for the Canada group were $-3.42$ and $-4,$ respectively, and the 95th percentiles were $-0.57$ and $-1,$ respectively. The 5th percentile of the bootstrapping mean and median differences in the number of months between the first mentions in the RAs and GT for the non-Canada group were $0.5$ and $0,$ respectively, and the 95th percentiles were $8.2$ and $3,$ respectively. These figures show that worldwide, most of the regional research studies appeared much faster than GT regional searches did, with the exception of Canada. According to the WHO dashboard^5^, the number of deaths in Canada reached their highest in April and May 2020, at more than 200 deaths per day. This may be the reason why “alberta” and “ontario” were first mentioned in GT in April 2020. We explored the reasons why the RAs’ results for Canada were later than those in GT, by dividing the keywords into a Canada group and a non-Canada group and considering the RAs that had at least one of the keywords in the Canada group and in the non-Canada group. We applied the CTC method to these two sets of RAs and found 134 and 326 CTC clusters, respectively. We include one of the CTC clusters in each set of the results in Table M4.1. The CTC cluster labelled CC is the cluster of keywords for the Canada group, while the CTC cluster labelled CN represents the cluster of keywords for the non-Canada group. The keywords of the CTC cluster CC were mainly about restrictive measures, such as social distancing, and some social support measures in the Canadian provinces. It appears that the RAs finally contained mentions of the status of COVID in Canada at that time––that is, the CC research studies comprised regional case studies of Canada. Because there were many reported deaths in April 2020, the research reported in the RAs was conducted at the peak time of the pandemic. In contrast with the Canada group, the CTC cluster CN reveals that the focus of the non-Canada group was on the origin of COVID and on preventive treatments. The researchers who took the initiative to study the origin of COVID may have had better preparation for the pandemic, and that may be why the time of the first mention of the virus’s origin in the non-Canada group was significantly earlier in the RAs than in GT. In contrast, the researchers for the Canada group may have underestimated the effects of COVID and may not have paid much attention to the origin of the illness.

In summary, with the exception of the group we designated as the Canada group, the RAs generally logged an earlier response to the regional status of COVID than GT searches did. In general, the RA researchers also studied the origin of the virus and were able to report on it quickly.

| 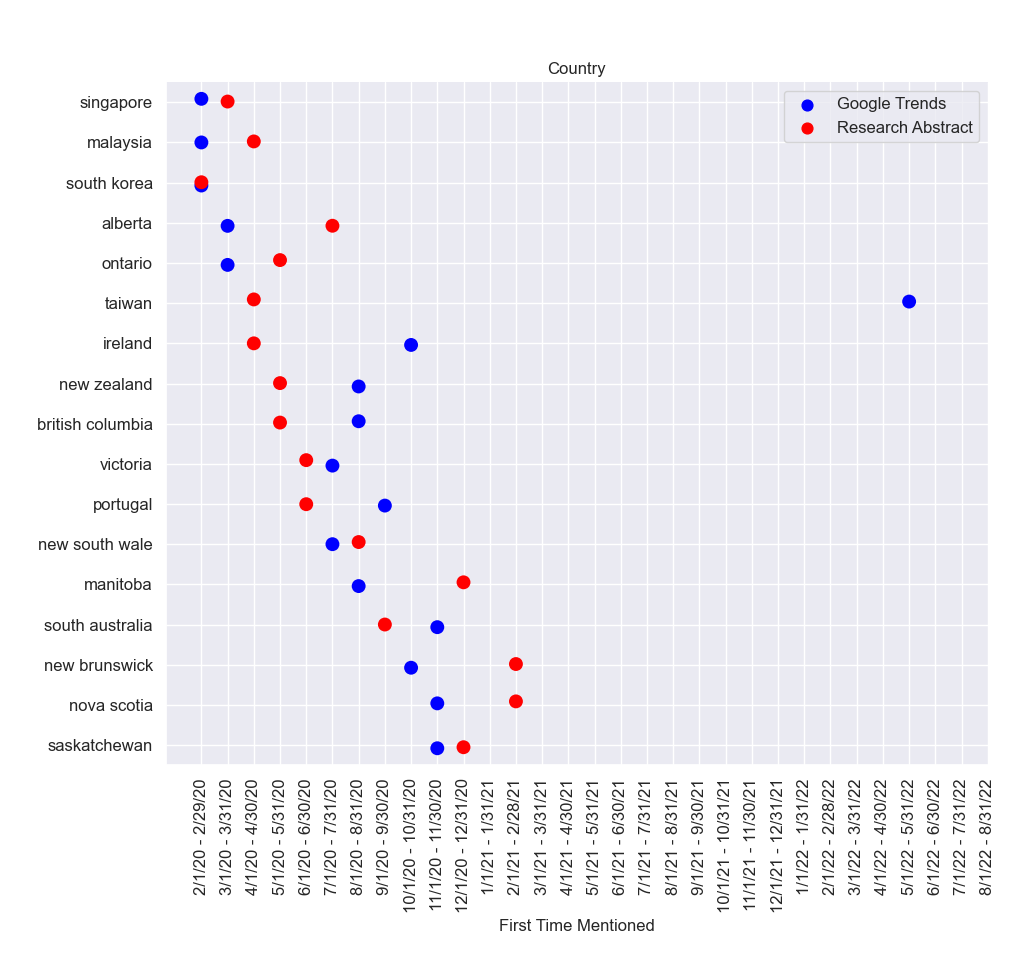 |
| --- |
| Figure M4.1. Time plot for the keywords/phrases under the category of Country. |

| **CTC Cluster ID** | **Noun Phrases** | **Rank Scores** |
| --- | --- | --- |
| CC | “ontario”, “experience”, “physical distancing”, “longitudinal”, “individuals”, “social support”, “canada”, “toronto”, “resilience”, “psychosocial”, “prior”, “ontario canada” | 552 |
| CN | “wuhan china”, “pathogenesis”, “cause”, “severe acute respiratory syndrome coronavirus”, “review discus”, “treatment strategy”, “treatment covid”, “syndrome”, “prevention treatment”, “effective treatment” | 2583 |
| Table M4.1. Identified CTC Clusters for Country. Total number of RAs (CC): 7,056, Total number of RAs (CN): 32,331. | | |

# Appendix M5 - Others

We combined into this group, which we called “others,” the rest of the keywords obtained by GT that could not be categorized in one of the five groups already discussed. The keywords for “others” are shown in Figure M5.1. The RAs contained most of those keywords earlier than GT did. The 5th percentile of the bootstrapping mean and median differences in the number of months between the first mentions of keywords in the two sources under the category “other” were $2.58$ and 1, respectively, and the 95th percentiles were $9.28$ and $6,$ respectively. In other words, the research articles in the RAs discussed these issues on average at least 2.58 months earlier, and 1 month (in terms of median) earlier, than the GT searches did.

We selected the keywords “smallpox” and “monkeypox” for further discussion, because they are more life-threatening than other issues. Monkeypox is a rare disease with symptoms that are similar to but milder than the symptoms of smallpox. However, monkeypox can cause death and is an emerging infection in Africa, and outbreaks of imported cases of monkeypox sometimes happen in other countries, including the United States. Therefore, public discussed both “smallpox” and “monkeypox” together. Both keywords were first mentioned in GT in August 2022, whereas the RAs included “smallpox” 24 months earlier than GT did. In fact, at the beginning of the pandemic, some researchers reviewed the historical success of smallpox eradication and tried to find comparable strategies to control COVID-19 (Heymann and Wilder-Smith, 2020). Some of those strategies were to allow only people who were monitored and had been vaccinated in contact with an infected person. This strategy seems also to have been used to fight COVID-19. Regarding the other keyword, “monkeypox,” The RAs included it one month earlier than Google Trends did. Researchers discussed the symptoms and threat of monkeypox (Lai et al., 2022) and also explored whether monkeypox had been exacerbated by the COVID-19 pandemic (Farahat et al., 2022).

| 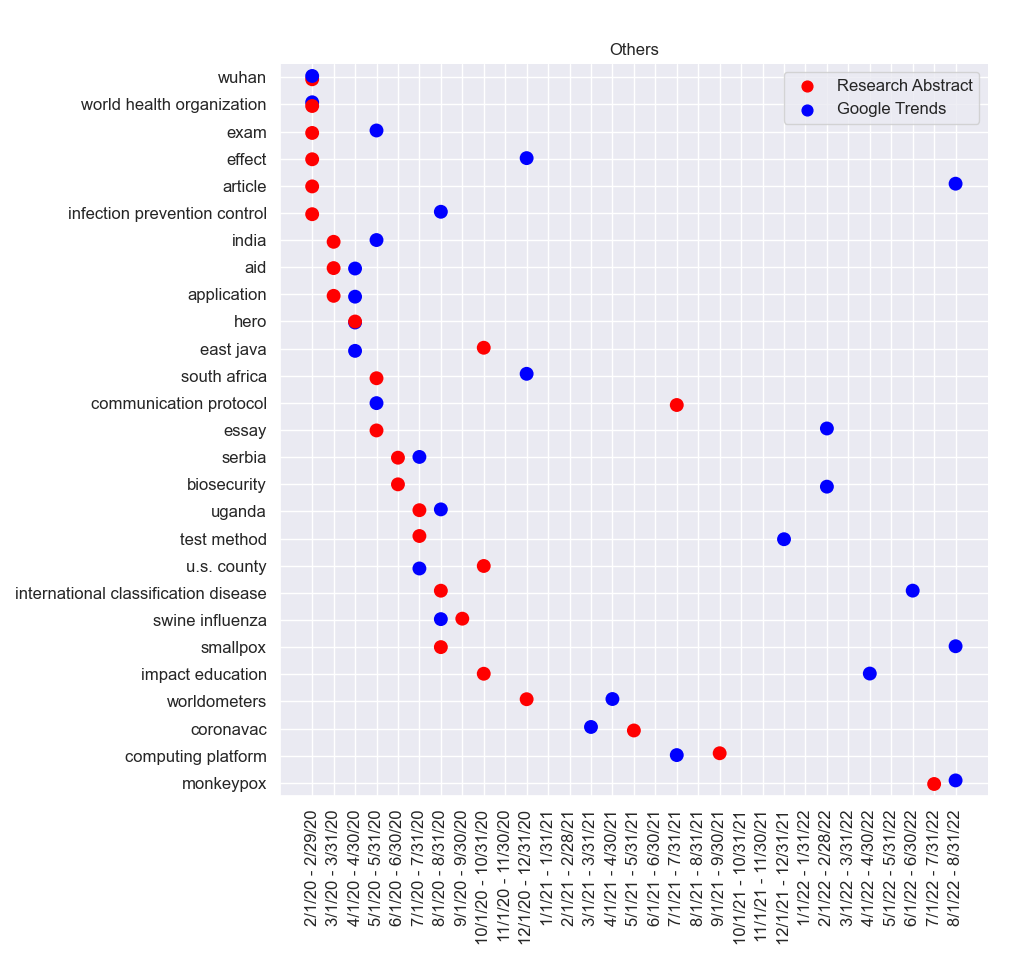 |
| --- |
| Figure M5.1. Time plot for the keywords/phrases under the category of Others. |
